# Evaluating the immune response in a murine cancer model between irreversible electroporation and an advanced biphasic pulsed electric field technology

**DOI:** 10.3389/fonc.2025.1592610

**Published:** 2025-06-24

**Authors:** Ebtesam H. O. Nafie, Chiara Pastori, Robert E. Neal

**Affiliations:** Medical Affairs, Galvanize Therapeutics, Inc., Redwood City, CA, United States

**Keywords:** PEF, aPEF, Aliya, non-thermal ablation, ablative immunostimulation, IRE

## Abstract

**Intoduction:**

Non-thermal ablation, including irreversible electroporation (IRE) and Aliya®, an advanced biphasic pulsed electric field (aPEF) technology, have emerged as effective tumor ablation approaches, particularly in sensitive anatomical locations. These methods not only ablate tumors but also may stimulate immune responses.

**Methods:**

This study compares the immunological impact of biphasic aPEF and IRE in a murine breast cancer model. Equal-sized tumor ablations were performed using both technologies, followed by analysis of cytokine profiles, immune cell populations, tumor growth, and overall survival.

**Results:**

aPEF induced a differentiated tumor microenvironment four days post-ablation compared to IRE, with greater intratumoral infiltration of T-cells, B-cells, increased M1 macrophages, and decreased myeloid-derived suppressor cells. Analysis of systemic circulating immunocytes 14 days post-ablation showed elevated levels of B-cells, CD4 and CD8 T-cells (including memory subpopulations) in the aPEF-ablated groups. aPEF also resulted in better control of ablated and contralateral tumor growth, leading to improved median survival.

**Discussion:**

This study demonstrates that the specific biphasic aPEF system evaluated here induces a stronger immunostimulatory effect and superior tumor control compared to IRE, supporting the notion that not all non-thermal ablation is equal, and each may be better suited to different objectives. Further clinical investigations into the potential for better clinical outcomes from this specific advanced pulsed electric field technology is warranted.

## 1 Introduction

Non-thermal ablation technologies, such as Aliya^®^ biphasic pulsed electric fields (aPEF) and irreversible electroporation (IRE), have provided a critical advance in focal ablation, especially for targets near vital structures (1, 2). These techniques use a series of electrical pulses delivered directly into tissue. When dose parameters are appropriately managed, it is possible to induce substantial volumes of tissue ablation while maintaining the integrity of the extracellular matrix (ECM), affording a superior safety profile over other focal techniques such as surgical resection, radiotherapy, and thermal ablation (3–5).

In addition to safer ablation, existing and emerging evidence has demonstrated the ability for these technologies to invoke a robust anti-cancer immune response in preclinical models and clinical responses (6–9). This is hypothesized to be related to the less injurious wound induction (3) and better preservation of tumor-specific antigens (10), as well as the release of various immunostimulatory damage associated molecular patterns (DAMPs) such as HMGB1 (9, 11).

IRE was described to induce more intact antigens, as well as improved synergy with aPD-1 immunotherapy than for cryoablation or heat (12, 13). IRE was also shown to invoke a pro-inflammatory tumor microenvironment and anti-tumor immunity in pancreatic cancer murine models (14). Clinical evidence shows that IRE has a generally safe profile, can provide local control, prolongs patient survival, and has the potential to trigger anti-tumor immunity (7, 15, 16).

Similarly, aPEF was shown in murine models to produce faster lesion resolution and favorable immunostimulatory phenotypes intratumorally and systemically, as well as better ablated and contralateral (unablated) tumor resolution than for thermal ablation of equivalent size (9). While published clinical evidence of immune stimulation from biphasic aPEF is in its infancy, a recent case series investigating a cohort of 17 patients progressing on their systemic therapy showed that 12 of 24 off-target (unablated) lesions previously progressing were unchanged or decreased in size after aPEF ablation at a median follow-up of 3 months (2). The authors conclude their findings suggest that the biphasic aPEF invoked a systemic effect capable of arresting the progression of these lesions despite their systemic therapy, which may be related to immunostimulatory properties. Further, a retrospective analysis demonstrated prolonged progression-free and overall survival for patients with Stage IV non-small cell lung cancer progressing beyond first line therapy that received aPEF ablation in addition to standard of care compared to a matched cohort (17).

While both IRE and aPEF have generally favorable safety profiles and immunostimulatory properties, there are distinct differences between the clinically available embodiments of these two technologies. IRE uses long-duration (50-100µs) pulses delivered between bipolar electrode arrays placed around the target area according to strict precision requirements and needs the addition of paralytic (18). Conversely, the Aliya aPEF System delivers biphasic energy in a manner that mitigates the need for paralytic and thus general anesthesia, permitting some cases to be performed under conscious sedation or local-only anesthetic (9). More notably, the reduced muscle contraction permits aPEF to be delivered with a monopolar electrode placed into the targeted zone for a given ablation activation to communicate with a distant dispersive pad, providing a more predictable ablation geometry.

Cell exposure to electric fields invokes a myriad of processes, some of which that ultimately result in cell death (19). Due to the distinct nature of the technologies, their waveforms, and their delivery routes, each technology invokes uniquely differentiated cell death processes and tumor microenvironment (TME) changes. These differences may impact the safety profile of the technologies and also the ability for each to invoke anti-cancer immunostimulatory effects.

This study evaluates the different downstream immunomodulatory effects of IRE and a biphasic aPEF waveform representing Aliya ablation. Specifically, the study delivers matched-size ablations with each technology to orthotopically implanted triple negative breast cancer tumor models, and systematically evaluates cytokine profiles, immunocyte populations in the TME and circulation, as well as the ability for each to clear ablated and off-target contralateral tumor inoculations.

## 2 Materials and methods

### 2.1 Study design and ablation cohorts

All animal studies were performed in accordance with the Institutional Animal Care and Use Committee (IACUC) protocol number 2023-05-01. The studies were carried out by an independent contract research organization (CRO), Bayside Biosciences, Inc., located in Santa Clara, CA.

### 2.2 Study overview

**Figure 1:**
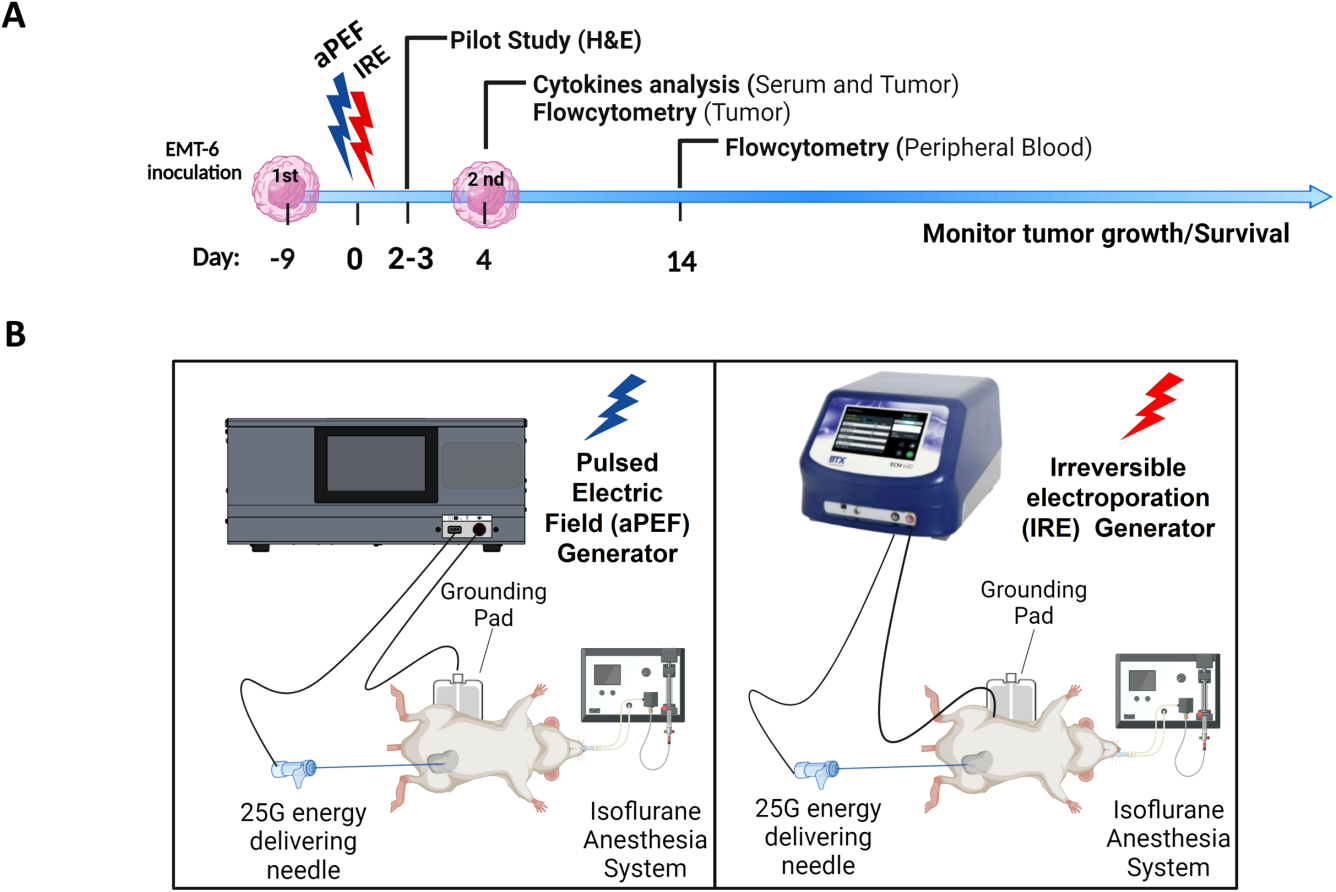
Experimental timeline and setup for ablation immunological effects comparison. **(A)** Timeline depicting the inoculation of EMT-6 tumor cells, followed by the administration of Pulsed Electric Field (aPEF) and Irreversible Electroporation (IRE) ablations. Cytokine level monitoring and immune cell dynamics through flow cytometry are indicated. **(B)** Schematic of the equipment setups for aPEF (left) and IRE (right) ablations, including the configuration of needles, electric field generators, anesthesia systems, and grounding pads.

The study design used for comparing the locoregional and systemic immunological effects of murine tumor ablation can be seen in **Figure 1**. Briefly, titrated doses of IRE or aPEF were delivered to inoculated tumors in a monopolar fashion to achieve matched ablation sizes targeting about 60% of the total tumor volume. This partial ablation was used to provide residual unablated tissue for evaluating the local cytokine environment and immune response. Four days following ablation, a subset of mice was euthanized to characterize TME cytokines and innate immune cell populations, while the remaining mice received a contralateral (untreated) inoculation of the same tumor cell line. Fourteen days post-ablation, blood was drawn to evaluate systemic adaptive immune cell populations. Mice were followed for tumor growth and survival until the end of the study.

### 2.3 Experimental groups

Tumors were grown in the mammary fat pad of mice. Once the tumors reached 5–7 mm in size (Day 0), the mice were randomly assigned by cage number to the experimental groups defined in **Table 1** and interventions were initiated.

**Table 1:** Summary of experimental groups by ablation modality and performed analysis.

Ablation group	Pilot H&E	Cytokines analysis	Flow cytometry (TME)	Flow cytometry (systemic)	Tumor growth, & survival
Sham	n=2	n=4	n=4	n=8	n=8
IRE	n=20	n=8	n=6	n=6	n=7
aPEF	n=3	n=6	n=6	n=10	n=10

#### 2.3.1 Pilot study: ablation size parameter matching

This study focused on determining whether the underlying technology-based effects to the TME and invoked cell death mechanisms between ablation approaches results in differentiated immunological and tumor responses. To avoid bias in local and distant tumor response based on total ablation volume, it is critical to ensure equivalent ablation volumes for both ablation technologies. Further, partial ablation of the inoculated tumors is necessary to properly delineate the benefits of the induced immune system on local tumor response.

To accomplish these conditions, two pilot experiments were performed prior to starting the study, whereby the same EMT6 tumor cell line was grown to 5–7 mm diameter tumors in the long dimension. Mice were then anesthetized and received waveforms consistent with IRE or aPEF, both using the same single needle electrode approach. A total of 100 IRE pulses at 100 µs were delivered, representing a typical IRE protocol (20), while the biphasic aPEF delivered 60 packets with a waveform that matched that from the commercially available Aliya system. Different voltages were used that were anticipated to approximately result in 60% tumor ablation based on prior tumor studies and preliminary bench testing *(data not shown)* using the formula (**Equation 1**) for ablation volume (in mm^3^):


(1) $$V=\frac{short\;axis\;\times \;long\;axis\;\times \;(short\;axis\;+\;5mm)}{2}$$

This equation incorporates any distortions from the shape of the tumor, and predicts the z-axis dimension as roughly the short-dimension ablation radius extending equally in all directions (including the z-axis), based on results provided in (4).

**Figure 2:**
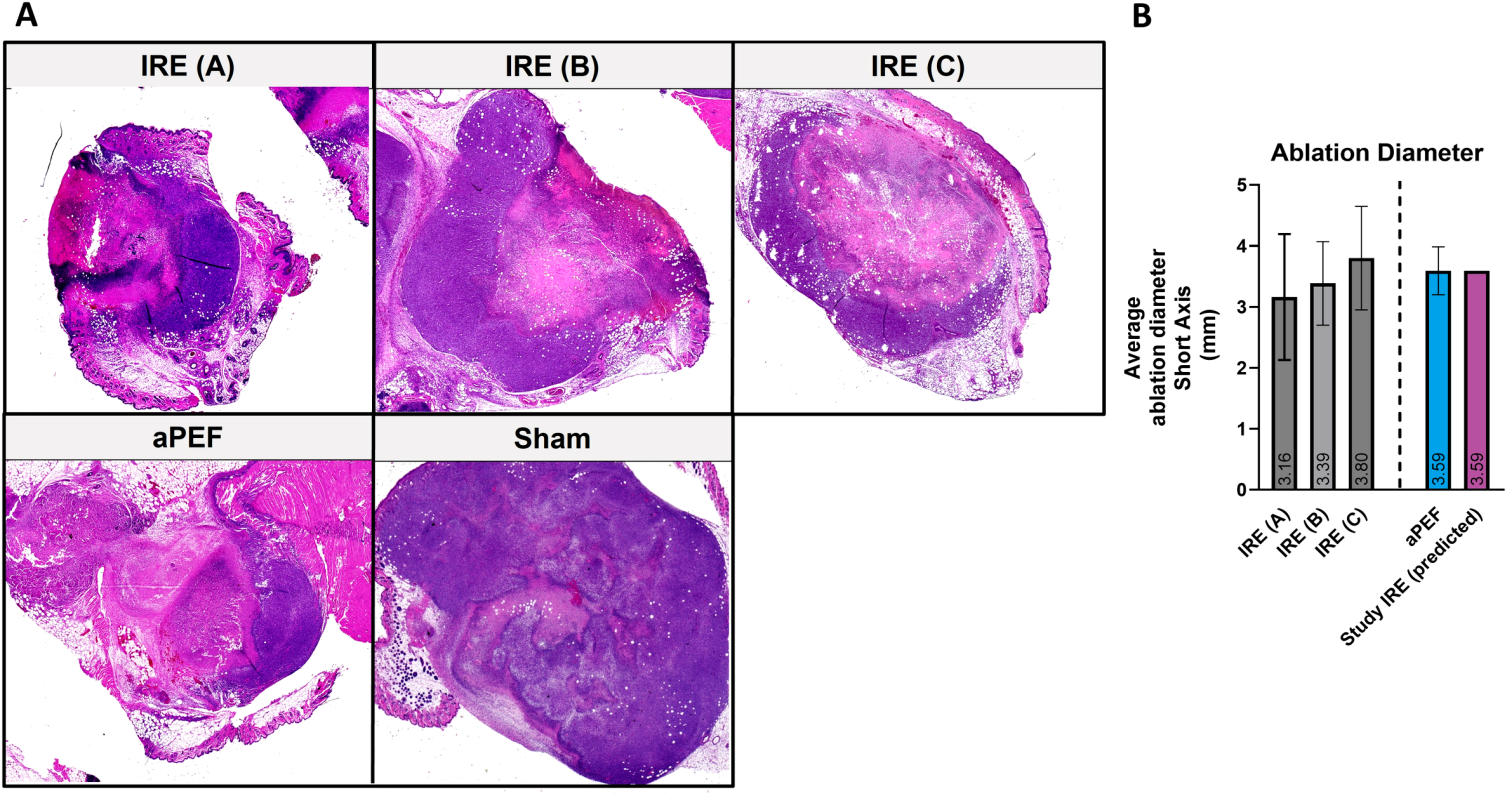
Comparative analysis of electroporation efficacy in tumor ablation in mice. **(A)** Histological sections of mice treated with low **(A)**, medium **(B)** and high-intensity **(C)** IRE (n=20), aPEF ablation (n=3), and Sham (n=2). **(B)** Bar graph illustrating the average short-axis length of tumor ablations in mice after ablations with varying intensities of IRE, aPEF, and the predicted IRE used in the study.

Two and three days following pilot ablation delivery, mice were euthanized, and residual tumors were harvested. Tumors were split along their midline in the long dimension (perpendicular to needle orientation) and processed for hematoxylin and eosin (H&E) histology (**Figure 2A**). Histology slides were scanned (PrimeHisto XE scanner, HistoView software), and measurements of the short axis diameter were made using ImageJ (NIH, MD, USA). These dimensions were used to determine the final voltage dose to use for the aPEF and IRE delivery to ensure adequately matched ablation volumes (**Figure 2B**). The final dose selected was noted to ablate approximately 60% of the tumor volume, characterized by a central zone of cell death, with a periphery of viable cancer cells.

### 2.4 Procedures

#### 2.4.1 Tumor inoculation

EMT6, a triple-negative breast cancer cell line purchased from the American Type Culture Collection (CRL-2755; Manassas, VA, USA) was authenticated through short Tandem Repeat analysis and used for all *in vivo* experiments. The cells were cultured in RPMI 1640 medium (MT10040CV, Corning, Manassas, VA, USA) containing 10% fetal bovine serum (092910154, MP Biomedicals, Irvine, CA, USA) and 1% Antibiotic-Antimycotic (15240–062, Gibco Life Technologies Corporation, Grand Island, NY, USA) at 37˚ C.

For both primary and contralateral tumor inoculations, 200,000 cells were inoculated in the 4^th^ mammary fat pad (left for primary, right for contralateral) of 6–8 weeks old female Balb/c mice (Charles River Laboratories, Wilmington, MA, USA). Contralateral inoculations were performed four days post-ablation to permit initiation of immune response in the ablated tumors, whereas synchronous inoculations would produce tumors requiring early euthanasia of the mice due to the rapid tumor growth rate of the model used.

#### 2.4.2 Mouse husbandry and tumor monitoring

Mice were housed together in ventilated cages (4–6 mice per cage) subject to light dark cycles. Food and water were provided ad libitum. Food, water, and bedding were changed and/or replaced two times per week. The well-being of mice was observed daily, and when an end point was reached, euthanasia was administered by CO_2_ asphyxiation immediately or within a few hours. Criteria for euthanasia included a tumor exceeding 1.5 cm in any direction, tumor preventing ambulation or ability to reach food and water for more than 24 hours, if tumors became severely ulcerated or abscessed, if mice became emaciated or lost 20% body weight, or if the mice showed signs of lethargy and labored breathing. If unexpected death unrelated to tumor burden occurred, the animal was excluded from data analysis.

Tumors were monitored and measured with electronic calipers three times per week, and tumor volumes were calculated according to the following formula (**Equation 2**):


(2) $$V=\frac{length\;\times \;width\;\times \;width}{2}$$

Tumor−volume data between day 0 and day 10 were censored because post−ablation edema and scar formation could distort size measurements during this interval. This blanking period did not alter final growth curves nor survival analyses for this study.

On day 4, a subgroup of mice was euthanized to evaluate the cytokine and immune cell populations in the TME. In the rest of the mice, a secondary contralateral tumor was inoculated on the opposite side of the 4^th^ mammary fat pad to monitor off-target (abscopal) effects of the ablations. On day 4 and day 14, retroorbital blood draws were collected for cytokine analysis (Day 4) and flow cytometric evaluation of the systemic immune response (Day 14).

### 2.5 Energy delivery

For both ablation technologies, mice were anesthetized with isoflurane (3% induction, 2% maintenance) and O_2_ inhalation using a chamber to induce anesthesia, and a nose cone for maintenance during the procedure (**Figure 1B**). The fur on the back of the anesthetized mouse was clipped and soaked with saline prior to placement on a dispersive return electrode (3M universal electrosurgical pad, cat#9165E, Saint Paul, MN, USA) that was coated with a high-conductivity electrode gel (Parker Laboratories, Enumclaw, WA).

To improve ablation volume matching and ensure consistency between ablated regions (e.g., central v. peripheral regions), the same electrode configuration was used, comprising a monopolar setup using the same needle electrode to deliver both forms of ablation. This approach was selected because the reduced voltages to target the 5 mm tumor permitted a monopolar delivery approach for IRE without excessive muscle contraction. Because the objective of this study was to evaluate differences in immune response between the two technologies, and both are amenable to bipolar or monopolar arrangements, a more accurate reflection of the distinctions between the core unique technologies required a standardized physical setup.

A custom-built 25-gauge needle (5.0 mm long electrical exposure) was inserted through the skin and centered in the tumor. The needle was connected to either a titrated energy Aliya generator for aPEF delivery or a BTX ECM 830 (Harvard Biosciences, MA, USA) for IRE delivery, as depicted in **Figure 1B**. Ablations were delivered at the settings for each system that was determined from the pilot study to result in size-matched partial ablation, targeting approximately 60% of the tumor volume. After completing ablation delivery, the needle was removed and mice received 500 µL of saline intraperitoneally and left to recover.

### 2.6 Immune response characterization

#### 2.6.1 Flow cytometry quantification of immune cell populations

Flow cytometry was used to quantify local and systemic immune cell populations at the timepoints depicted in **Figure 1A**. Approximately 200 μL of blood was collected via retroorbital bleed in EDTA-containing tubes on day 14. Red blood cells were lysed using 1 ml of red blood cells Lysis buffer (420201, Bio-Legend, San Diego, CA, USA). Blood was mixed briefly to resuspend the remaining cells (peripheral blood mononuclear cells, PBMCs), incubated for 15 min at room temperature, and centrifuged. The PBMCs were resuspended in FACS buffer (B51503, Beckman Coulter, Brea, CA) and incubated in Fc blocking buffer (156604, anti-mouse CD16/32 antibody, Biolegend, San Diego, CA, USA) at room temperature for 15 min.

The cells were divided into individual tubes for the respective cell type analyses, suspended in 50 μL of staining buffer (420201, Biolegend, San Diego, CA, USA), and stained for the designated cell type. The antibodies used are listed in **Supplementary Table 1**. After staining, the samples were washed and fixed with 1% paraformaldehyde (J61899; Alfa Aesar, Ward Hill, MA, USA) before analysis using a CytoFLEX3 flow cytometer (Beckman Coulter Life Sciences, Brea, CA, USA). The gating strategy for the analysis is provided in **Supplementary Figure 1** for tumor analysis and **Supplementary Figure 2** for blood analysis. Additionally, the marker profile for identifying cell populations in tumor cell populations is detailed in **Supplementary Table 2**, while the marker profile for blood cell populations is outlined in **Supplementary Table 3**.

#### 2.6.2 Cytokines analysis

Serum and tumor cytokines analysis was performed on samples collected on day 4. Tumors were excised from euthanized mice and mechanically disrupted using scissors. The tissue fragments were combined with RIPA lysis buffer, and sonication was performed to enhance cell disruption and facilitate the release of intracellular cytokines. The lysed material was then centrifuged at maximum speed (≥15,000 × g) at 4°C to pellet cell debris. The supernatant, containing intracellular and extracellular tumor proteins, was carefully collected for tumor cytokine analysis.

Serum was obtained from blood collected retro-orbitally. The blood was allowed to clot at room temperature for 30 minutes. The clotted blood was subsequently centrifuged, and the resulting supernatant (serum) was transferred to a new tube for use in cytokine analysis.

The analysis of cytokines was performed by Eve Technologies (Calgary, Canada) for quantification of 32 cytokines, particularly those focusing on injury and pro-inflammatory processes (VEGF, Eotaxin, G-CSF, GM-CSF, M-CSF, IL-1α, IL-1β, KC, LIF, LIX, MCP-1, MIP-1α, MIP-1β, MIP-2, LIF, LIX, RANTES), Th-1 immune responses (IL-2, IL-10, IFN-γ, TNFαa, IP-10, MIG, IL-12p40, IL-12p70), and Th-2 immune responses (IL-3, IL-4, IL-5, IL-6, IL-10, IL-13, IL-17). To quantify cytokines, Eve Technologies utilizes the Luminex^®^ xMAP^®^ technology and the results are reported as fluorescence intensity (FI) values.

### 2.7 Statistical analysis

All statistical analyses were performed using Prism software (GraphPad, CA, USA). Differences between means of two unpaired samples were analyzed using Student’s t-test. Cytokine analyses were conducted using multiple comparison t-tests, with q-values to control the false discovery rate. Flow cytometry data were analyzed using one-way ANOVA. The significance of overall survival was evaluated using Kaplan-Meier curves and assessed with the Log-rank test, while the median survival was analyzed using the Gehan-Breslow-Wilcoxon test. Tumor volume comparisons between two groups were performed using Student’s t-test. Statistical significance was set at p< 0.05.

A formal power analysis was not performed for this study. Group sizes were based on prior experience with similar models and designed to balance statistical rigor with ethical considerations for animal use. The observed effect sizes and variability were consistent with previously published work, supporting the adequacy of the sample size for exploratory analysis.

### 2.8 Data integrity

To reduce bias, data management was handled by third parties, with blinding implemented as necessary. Mice were randomly allocated to ablation groups based on their cage numbers. Mouse husbandry, tumor inoculations, tumor measurements, euthanasia decisions, and collection of biological samples for analysis were overseen by an independent contract research organization (CRO), Bayside Biosciences (Santa Clara, California). Cytokine quantification was conducted by Eve Technologies (Calgary, Canada). All CROs were blinded to the ablation conditions of the mice.

## 3 Results

### 3.1 Ablation size evaluation

In the pilot study, murine tumor ablated zones four days following ablation via H&E staining revealed that low (A), medium (B), and high (C) IRE parameters as well as aPEF induced ablation zones measuring 3.16 mm, 3.38 mm, 3.80 mm, and 3.59, respectively (**Figures 2A, B**). An interpolated voltage between that used for IRE B and IRE C was determined to closely match the ablation size from aPEF, which is represented in **Figure 2B** labeled as “IRE Study, Predicted,” and was used in the main study.

Based on the ablation dimensions, the calculated ablation volumes for both PEF and IRE using the parameters applied in the study resulted in 66.0 mm³ and 80.8 mm^3^ for aPEF and IRE, respectively. Using the measured tumor volumes in the experiment with these calculated ablation volumes, the percentage of tumor ablation was calculated to be 57% and 61% for the aPEF and IRE groups, respectively, confirming consistent absolute and relative percentage ablation in the study.

### 3.2 Cytokine expression

Levels of 32 cytokines in mouse serum and tumor tissue four days post-ablation for all ablation groups are provided in **Supplementary Tables 4**, **Supplementary Table 5**. Statistically significant differences in cytokine and chemokine between aPEF and IRE are summarized in **Table 2**.

**Table 2:** Cytokine levels (fluorescence intensity, FI) in tumor and serum 4 days post-ablation.

Cytokine	Sham (FI)	aPEF (FI)	IRE (FI)	aPEF vs IRE (p-value)
Tumor				
Eotaxin	6168.0	1714.0	6420.0	0.002
IL-1β	61.0	30.3	52.3	0.051
IL-2	83.8	19.6	62.3	0.007
IL-7	44.8	24.6	33.3	0.022
IL-10	48.7	25.9	48.6	0.039
IL-12	121.9	64.5	85.9	0.046
IL-13	73.7	36.9	57.8	0.045
CXCL1	656.6	1020.0	480.9	0.026
CXCL5	108.7	299.3	109.4	0.014
CXCL9	8306.0	4199.0	7782.0	0.018
CCL3	67.4	727.4	235.0	0.056
Serum				
IL-1β	11.2	9.9	7.8	0.012
TNFα	20.0	16.9	13.6	0.015

There were 11 significantly different cytokine levels in the post-ablative TME between the ablation modalities, reflecting their differentiated impact on the cells and TME. For the cytokines CXCL1, CXCL5, and CCL3, the levels in aPEF were higher than IRE. These cytokines are generally associated with innate immune activation and immune cell recruitment, particularly neutrophils and macrophages. Conversely, cytokine levels of Eotaxin, IL-1β, IL-2, IL-7, IL-10, IL-12, IL-13, CXCL9, were lower for aPEF than IRE. Notably, IL-2 and IL-12 are key cytokines that stimulate adaptive immune responses by promoting T cell proliferation and cytotoxic activity, while others such as IL-10 and IL-13 are more commonly associated with anti-inflammatory functions. This suggests that IRE may effectively enhance cytokine-driven adaptive immune activation. Additionally, IL-7 supports T cell survival and homeostasis, while CXCL9 plays a role in effector T cell recruitment to the tumor. IL-1β, although pro-inflammatory, can contribute to tumor progression or immune suppression and Eotaxin is generally linked to eosinophil recruitment and type 2 immunity. In serum, the pro-inflammatory cytokines IL-1β and TNFα were reduced for the IRE group compared to aPEF (p= 0.012 and 0.015, respectively), while both were lower than that of sham procedure.

### 3.3 Flow cytometry immune cell populations

#### 3.3.1 Intratumoral innate immune cells

**Figure 3:**
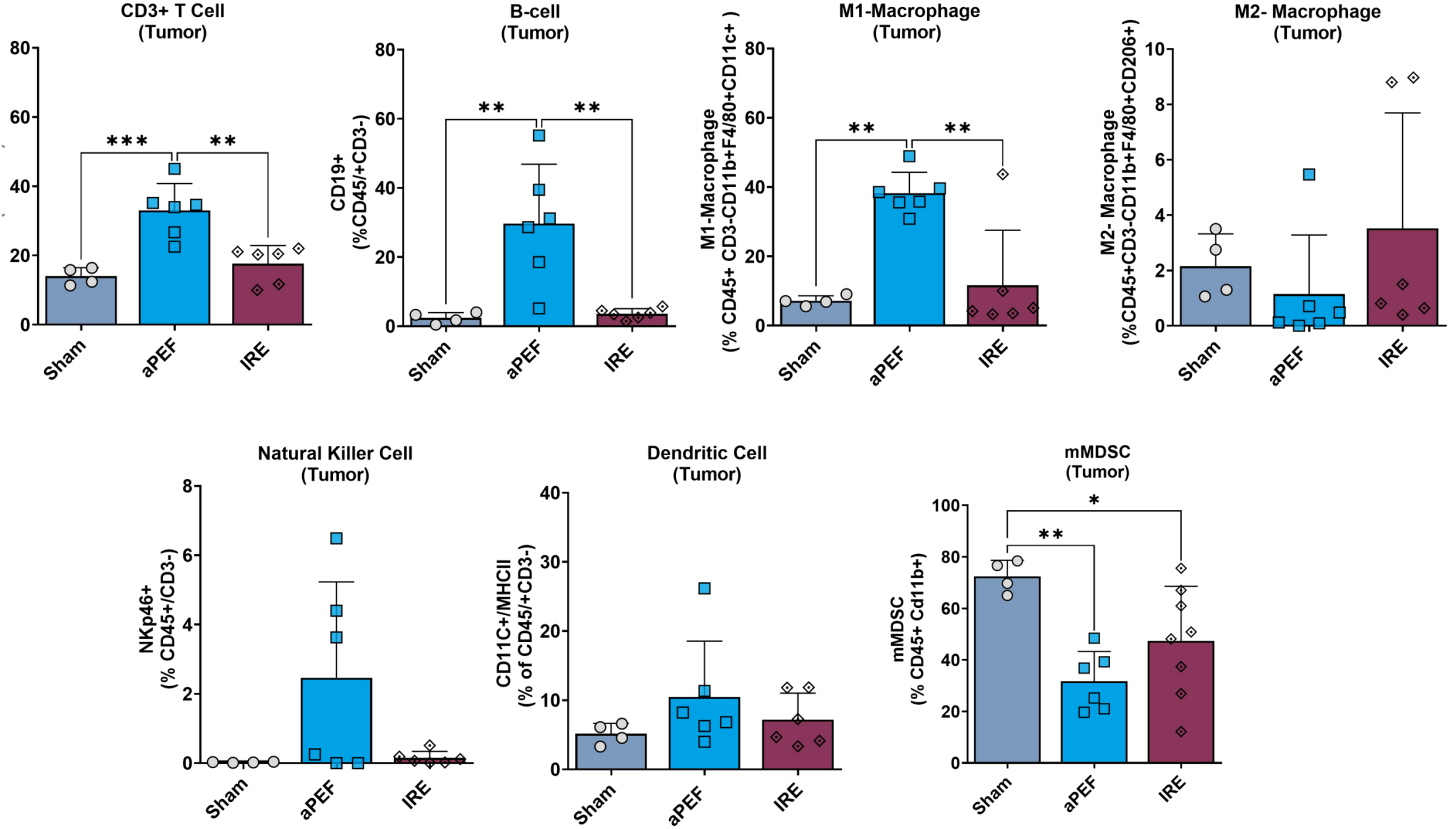
Intratumoral immune cell infiltration 4 days post-ablation. This figure displays the percentages of various immune cells within the tumor microenvironment after the treatment (n=4), aPEF (n=6) or IRE (n=6) ablation modalities: CD3+ T cells, CD19+ B-cells, M1-macrophages, M2-macrophages, Natural killer cells, Dendritic cells, and Myeloid-derived suppressor cells (mMDSC). The y-axes reflect the number of cells of the specific population or subpopulation, as a percent of the parent population indicated in parentheses. Data represents individual samples with mean ± standard deviation indicated by bars. Statistically significant differences between groups are indicated with asterisks (*p< 0.05, **p< 0.01, ***p< 0.001).

Tumor tissue harvested from mice euthanized four days post-ablation showed marked differences in innate and adaptive immune cell populations between ablation groups at this short-term timepoint (**Figure 3**). Statistically significant increases were observed in CD3+ T-cells (p=0.002) and CD19+ B-cells (p=0.003) in the aPEF group compared to sham and IRE groups. In addition, innate immune modulation was evident with a significant increase (p=0.002) in M1 macrophages (CD11b+, F4/80+ CD11c+) for the Aliya group. These findings contrast with the lack of significant differences between the sham and IRE groups for these populations.

Furthermore, a notable reduction in the immunosuppressive myeloid-derived suppressor cells (mMDSCs: CD11b+, Ly6C+, Ly6G-) was observed in both aPEF (p=0.004) and IRE (p=0.045) ablations relative to the sham control.

#### 3.3.2 Systemic adaptive immune cells

**Figure 4:**
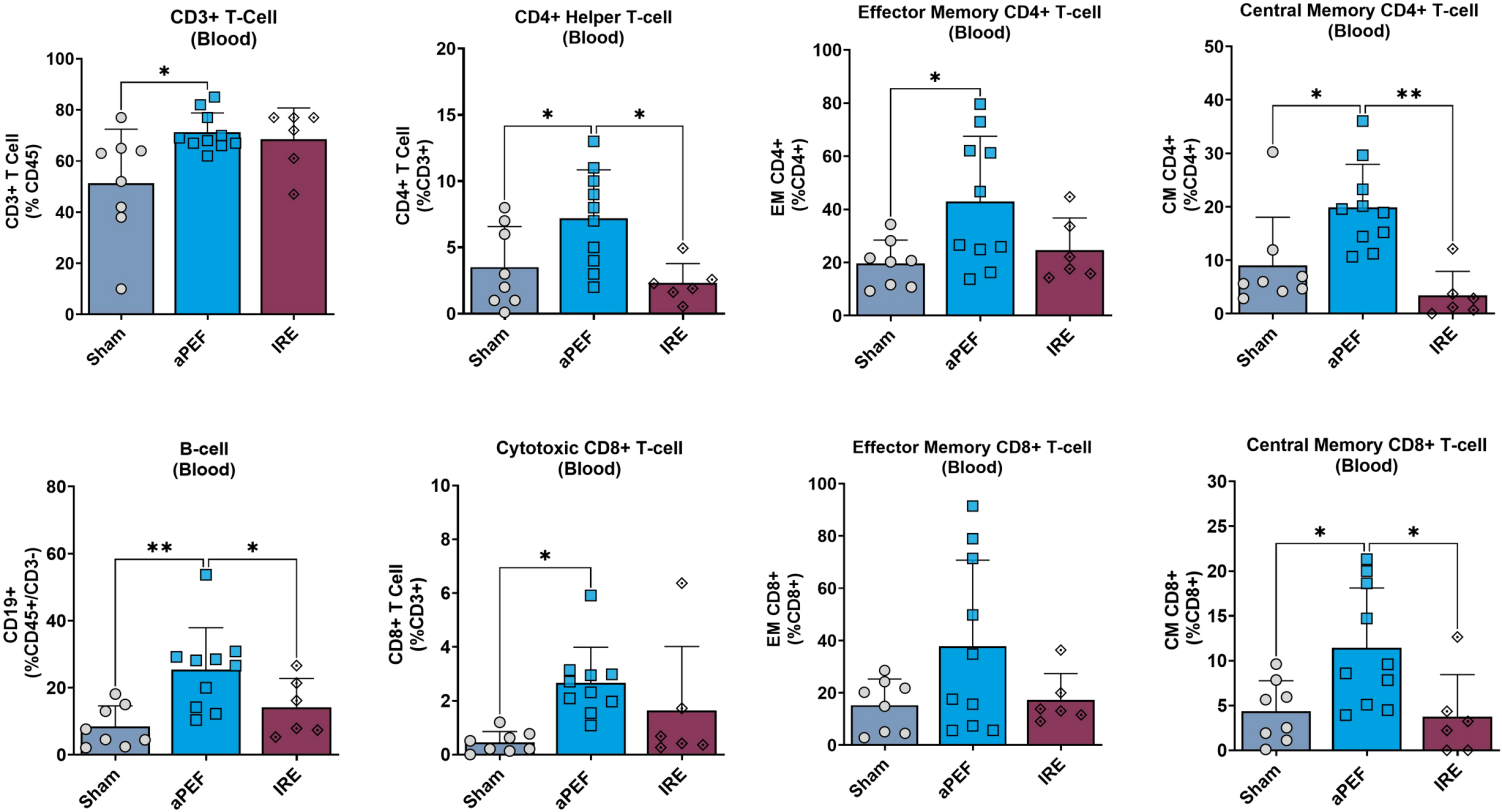
Systemic immune cell profile 14 days post-ablation. This figure presents the systemic immune cell profile in the blood at 14 days post-ablation ablation: CD3+ T cells, CD4+ helper T cells, Effector memory CD4+ T cells, Central memory CD4+ T cells, B cells, Cytotoxic CD8+ T cells. Effector memory CD8+ T cells, and Central memory CD8+ T cells. Each panel compares immune cell frequencies among three groups: Sham (control, n=8), aPEF (n=10), and IRE (n=7). The y-axes reflect the number of cells of the specific population or subpopulation, as a percentage of the parent population indicated in parentheses. Data represents individual samples with mean ± standard deviation indicated by bars. Statistical significance is noted with asterisks where *p< 0.05, **p< 0.01.

Longer-term analysis of peripheral blood showed several significant phenotypic changes in adaptive immune cell populations and subpopulations (**Figure 4**). aPEF had significant increases in overall levels of CD3+ T-cells (CD45+/CD3+) relative to sham (p= 0.022), while there was no difference for the IRE group to either Sham or aPEF. Further, aPEF led to statistically significant increases in CD4+ Helper T-cells (CD45+/CD3+/CD4) compared to sham (p=0.037) and IRE (p=0.016). Within the subpopulations, aPEF induced significant increases compared to Sham (p=0.014) and IRE (p=0.001) central memory (CM) CD4+ Helper T-cells (CD45+/CD3+/CD4+, CD44+/CD62L+). as well as a significant increase in effector memory (EM) CD4+ Helper T-cells (CD45+/CD3+/CD4+/CD44+/CD62L-) relative to sham (p=0.034). Regarding CD8+ cytotoxic T-cells, aPEF induced significant increases in total CD8+ T-cells (CD45+/CD3+/CD8+) relative to Sham (p=0.013). Additionally, central memory (CM) CD8+ T-cells (CD45+/CD3+/CD8+/CD44+/CD62L+) showed a significant increase in the aPEF group relative to Sham (p=0.030) and IRE (p=0.031). Finally, aPEF was found to induce statistically significant increases in B-cell populations relative to Sham (p=0.001) and IRE (p=0.030). Conversely, IRE was not found to invoke significant increases in any T-cell nor B-cell CD4+ nor CD8+ in all subpopulations evaluated relative to Sham. These findings reflect phenotypic shifts but did not determine whether the expanded CD4^+^/CD8^+^ subsets were tumor−specific or functionally cytotoxic.

### 3.4 Tumor response characteristics

#### 3.4.1 Primary and secondary tumor growth

**Figure 5:**
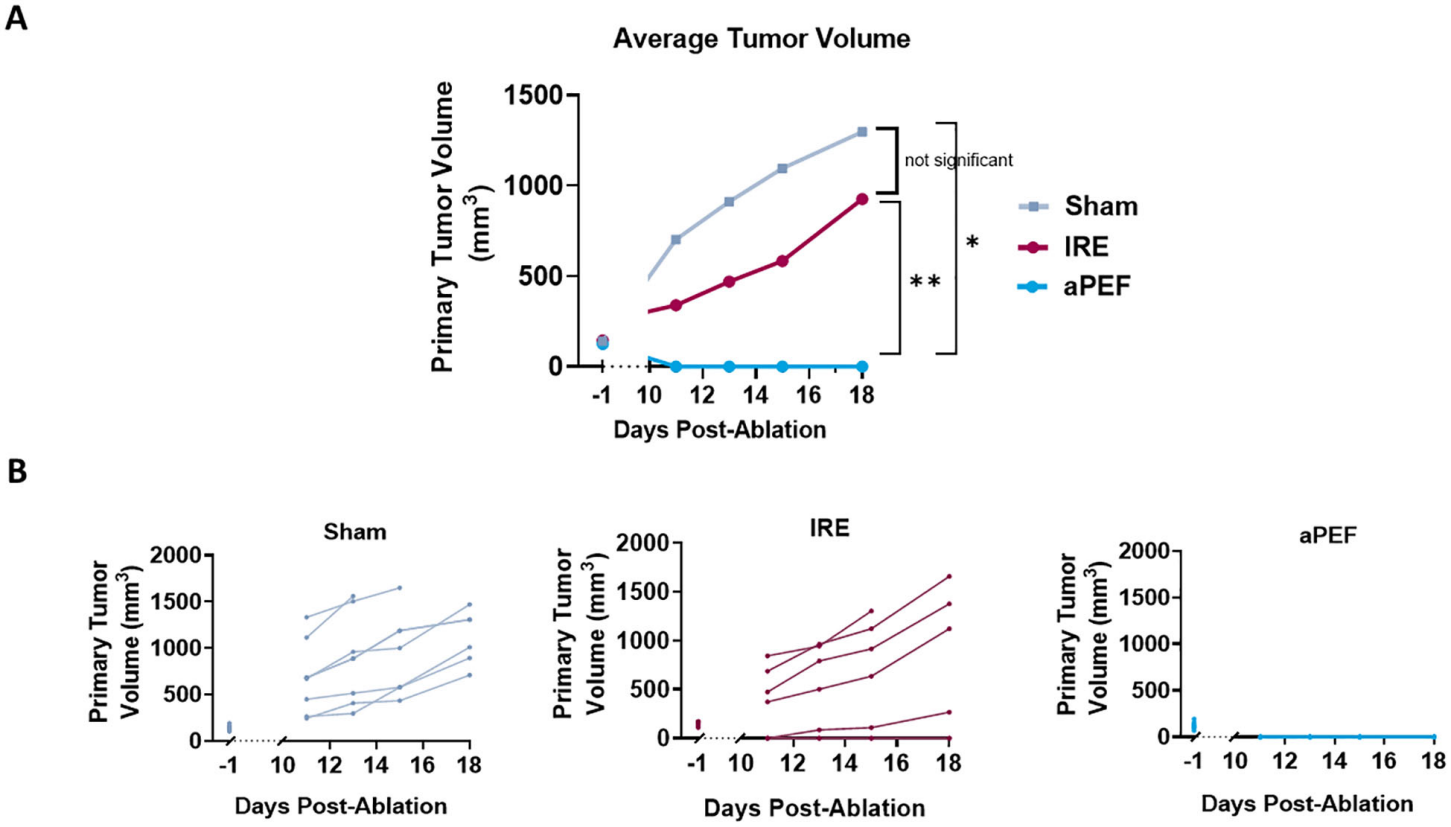
Impact of ablation on primary tumor growth volume. **(A)** Average tumor volumes plotted over a 18-day period post-ablation. Groups include Sham control (n=8), IRE (n=7), and aPEF (n=10). **(B)** Individual growth curves for tumors under different conditions. The graphs detail tumor response for each group, segmented into five separate panels Sham, IRE and aPEF illustrating the variability and response patterns within each group. Tumor−volume data collected between day 0 and day 10 were censored, because post−ablation edema and early scar formation could distort size measurements during this interval. Statistical significance is denoted by asterisks (*p< 0.05, **p< 0.01) at specific time points comparing effects.

While immune differences were observed, it is important to determine whether the differences are of sufficient magnitude to invoke different ablated and off-target tumor outcomes. Tumor volumes were compared until day 18, at which point mice began being euthanized due to tumor burden. The mean tumor volumes at this final time point were as follows: Sham 1823 mm^3^, aPEF 0 mm^3^, IRE 210 mm^3^ (**Figures 5A, B**). Statistical analysis of day 18 tumor volumes revealed significant differences between aPEF and the Sham and IRE groups (p = 0.008, 0.002, respectively), while comparisons between IRE and Sham did not show a significant difference (p =0.2).

**Figure 6:**
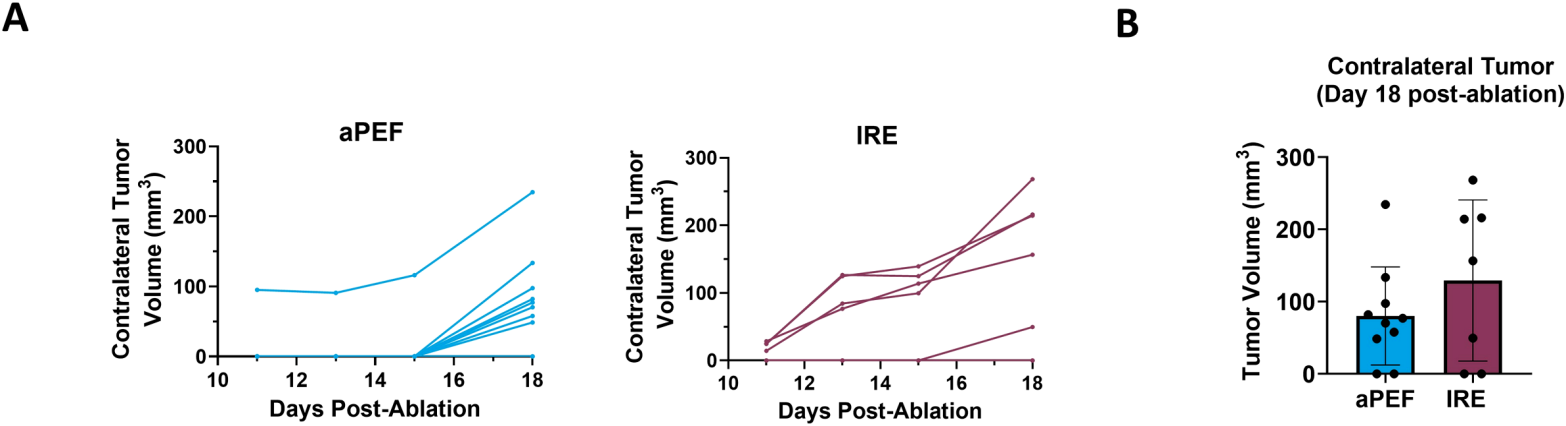
Impact of ablation on contralateral tumor volume. **(A)** Individual growth curves for contralateral tumors (inoculated 4-days post-ablation) under different ablation conditions: aPEF (n=10) and IRE (n=7). **(B)** Average contralateral tumor volumes plotted on Day 18 post-ablation. Sham group contralateral data was excluded due to the early euthanasia of most Sham animals, due to excessive primary tumor burden.

Contralateral tumor growth was tracked to evaluate off-target benefits that may be invoked from immunostimulation by the ablation technologies. The graphs in **Figure 6A** show the growth curve for contralateral tumors in the aPEF and IRE groups while the bar plot in **Figure 6B** illustrates the average tumor volumes of untreated contralateral tumors on Day 18 post-ablation. While the average volume in the aPEF group (80.2 mm³) trended lower compared to the IRE group (129.3 mm³), the difference was not statistically significant.

#### 3.4.1 Survival

**Figure 7:**
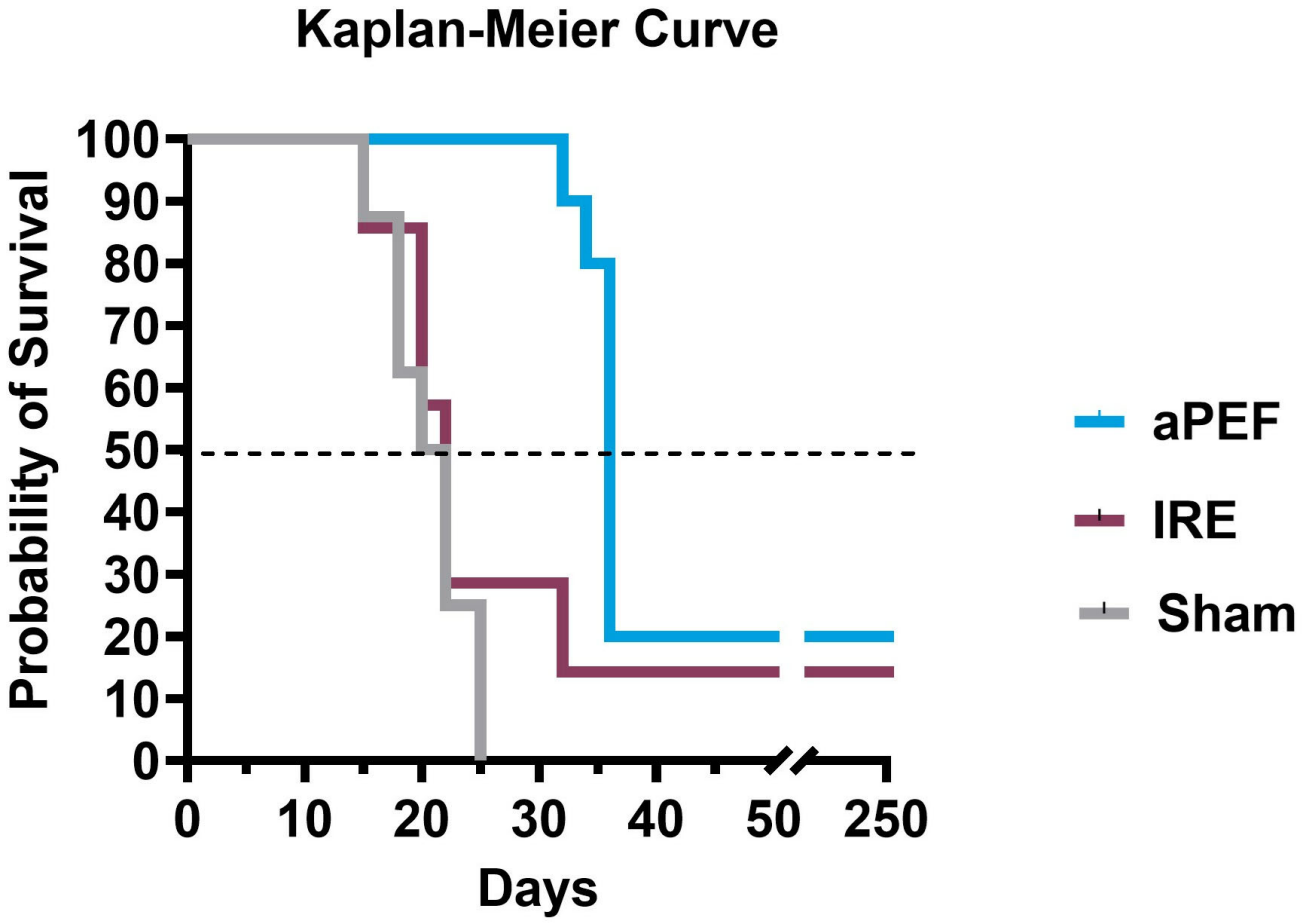
Survival outcomes post-ablation. Kaplan-Meier survival curves show the probability of survival over 250 days post-ablation, along with tabulated median survival following ablation. Statistical analysis using the Wilcoxon test was performed to determine differences in median survival rates. Sham group (n=8), aPEF (n=10) and IRE (n=7). Statistical significance: p < 0.05 (), p < 0.01 (*), ns, not significant.

Survival analysis via Kaplan-Meier up to 250 days post-ablation (**Figure 7**) demonstrated that none of the Sham-treated mice survived beyond day 25. Median survival across groups were 20 days for Sham control, 22 days for IRE and 36 days for mice ablated with aPEF. At the end of the study, survival was 17% and 20% for the IRE and aPEF ablated mice, respectively. The log-rank test did not reveal statistical differences in overall survival between aPEF and IRE. The Gehan-Breslow-Wilcoxon test identified a statistically significant difference in median survival between aPEF and IRE (p= 0.004) (**Table 3**).

**Table 3:** Median survival comparison between ablation modalities.

Group	Median Survival (days)	p-value
Sham	21	Sham v. aPEF: p<0.0001
aPEF	36	aPEF v IRE: p=0.0043
IRE	22	IRE v. Sham: p=0.5218

## 4 Discussion

**Figure 8:**
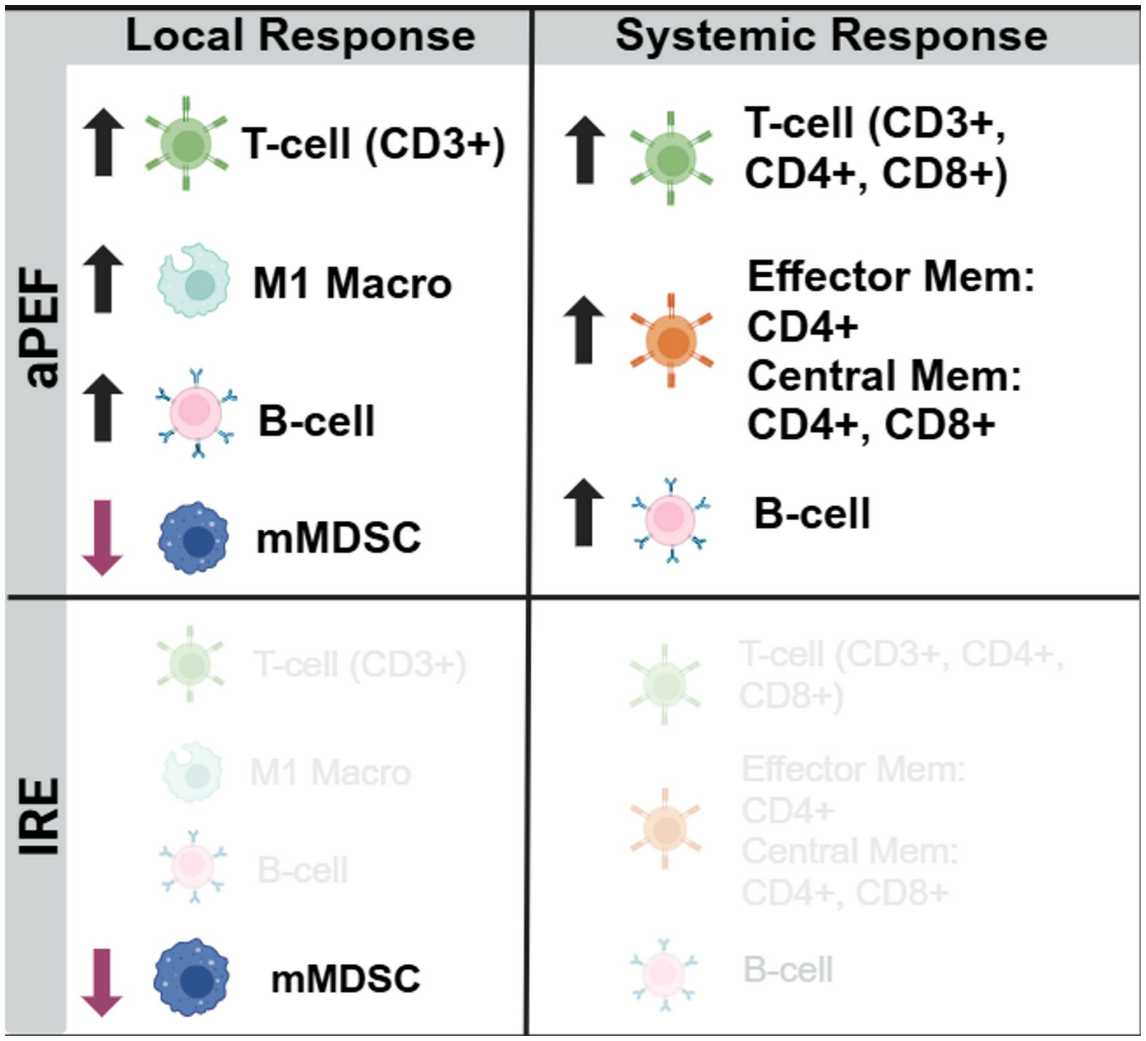
Immunocyte changes. Summary depiction of statistically changed immune cell populations relative to control. Schematic summary comparing immune modulation following aPEF treatment versus IRE. aPEF induced increased infiltration of CD3⁺ T cells, M1-like macrophages, and B cells locally, along with decreased monocytic myeloid-derived suppressor cells (mMDSCs). Systemically, aPEF enhanced total T cells (CD3⁺, CD4⁺, CD8⁺), memory T cells (effector and central), and B cells. IRE elicited minimal detectable immune changes in both local and systemic compartments.

**Figure 9:**
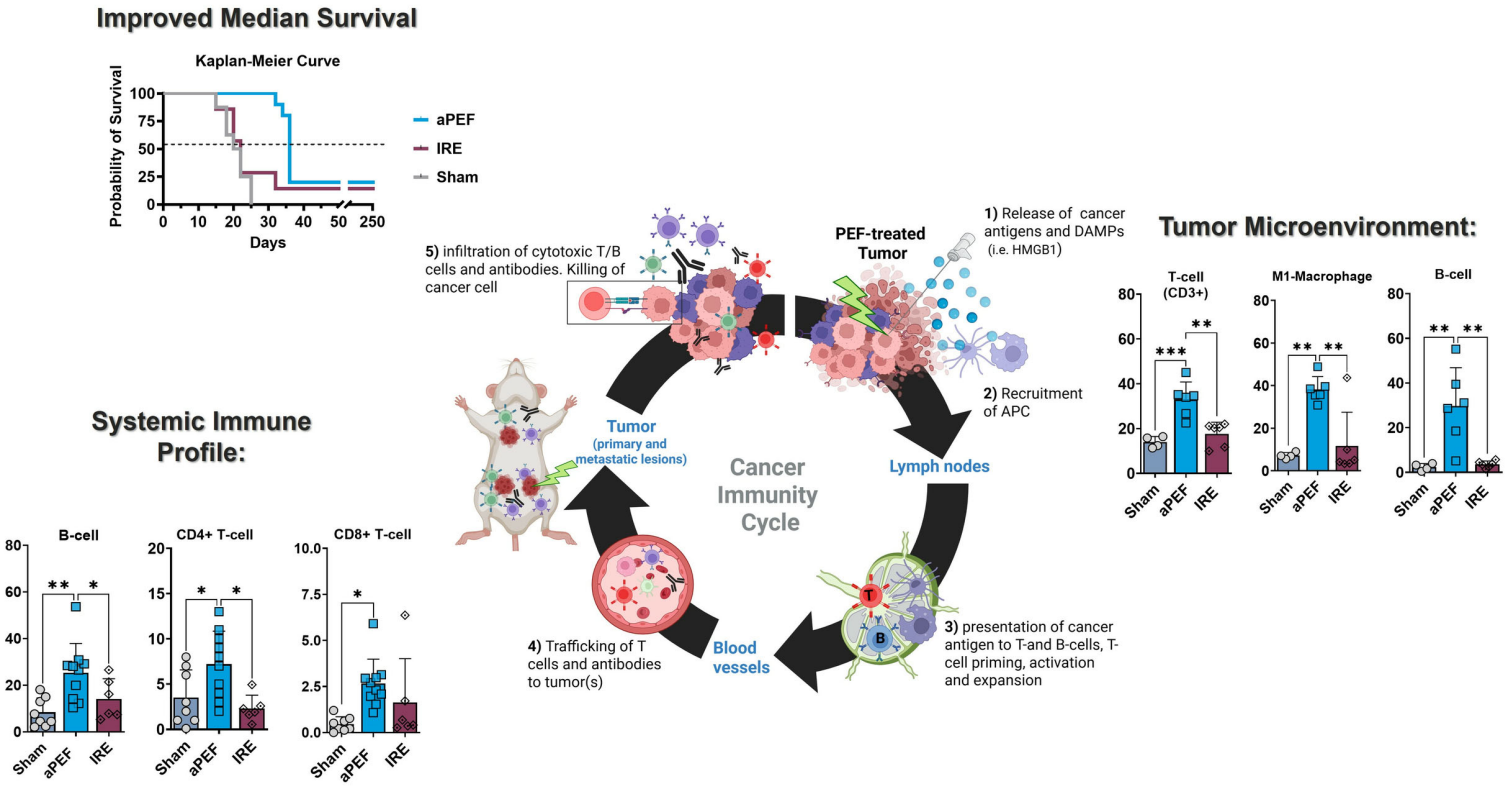
Comparative impact of aPEF and IRE on immune dynamics and tumor response. This figure highlights key findings, including enhanced immune activation (CD19+ B-cells, CD3+ T-cells, M1 macrophages) and improved survival with aPEF. Components include (1): Tumor microenvironment bar charts comparing dendritic cells and M1 macrophages; (2) Circulating immune cell profiles for CD19+ B-cells, CD3+ T-cells, and CD8+ T-cells; (3) Kaplan-Meier Curve showing survival probability. Statistical significance (*p< 0.05, **p< 0.01) underscores the superior immunostimulatory effects of aPEF.

This study delivered size-matched partial ablation of EMT6 orthotopic murine breast tumor models with IRE or an advanced biphasic form of PEF (aPEF) to examine the capacity for each to invoke anti-cancer immune responses capable of controlling residual ablated tumor growth as well as controlling contralateral off-target (i.e., “abscopal”) tumors. Despite equal percent volumes of ablation, the IRE group exhibited mild to no measurable changes in cytokines and immune cell populations relative to sham control, resulting in moderated ablated tumor growth decrease and no median survival benefit. Conversely, the aPEF group demonstrated a different post-ablative TME than IRE via significant differences in 11 cytokines and increases in multiple innate immune cell populations four days after ablation, along with pronounced increases in adaptive immune cell populations systemically 14 days post-ablation (**Figures 8**, **9**). These significant immunomodulatory differences resulted in statistically significant differences in primary tumor control and improved overall median survival for the aPEF ablation (**Figure 9**).

An important consideration in the study design was the decision to use the standardized monopolar electrode arrangement for both technologies, whereas most IRE delivery requires bipolar electrode arrangements. This decision was made for several reasons. bipolar electrode arrangements generally adjust voltage to compensate for different needle separations, attempting to maintain a consistent voltage-to-distance ratio (e.g., 1500 V/cm) (21). For 5mm tumors, a typical bipolar needle separation of 3–4 mm would mean that even a 1 mm variation in separation distance at the needle tips would deviate this ratio by 25-33% from the target, causing widespread variability in ablation volumes. Further, it was shown in (22) that even when voltage-to-distance ratios are maintained, the ablation shape and volume still vary significantly. Both of these conditions were deemed to inject too much variability into the study, as matching the ablation volume and region was critical to comparing the effects and downstream immunological outcomes. Future studies may compare the technologies using other electrode arrangements to determine the relative influence of physical electrode geometry on immunological stimulation.

Overall, 11 statistically significant differences between aPEF and IRE were found in the post-ablative PATME was 11 changes, serum was 2 changes, which may reflect more modest structural tissue damage and wound healing processes for aPEF. This finding is consistent with prior investigations on aPEF relative to thermal ablation (9).

This study did not observe meaningful changes in immune cell populations for the IRE group, which contrasts with several prior papers (23, 24). In He et al., 2020, it was shown that IRE can trigger significant immune cell infiltration, particularly CD8+ T cells and macrophages which were absent in this study. The prior study used a murine pancreatic cancer model with a different immune profile and tumor microenvironment, where differences in relative ablation volume, tumor type, immune microenvironment, and electrode design may have resulted in different findings. Further, a previous subcutaneous study on IRE using a 2-plate electrode system intending partial ablation of subcutaneous breast tumors showed slowed tumor growth, improved survival, and subsequent rechallenge rejection for immunocompetent mice but not immunodeficient mice (6).

Another difference between the study here and the literature was the use of 60% partial ablation, rather than the 80% cited in other studies (9, 25). This difference likely results from the variable nature of *in vivo* trials, whereby the rapid tumor growth coupled with small variances in cancer inoculation conditions (confluence, viability, actual number of inoculated cells, timing) may have a meaningful influence between average tumor volume at the time of ablation. This further highlights the need for direct prospective comparisons under identically matched conditions for each study, as opposed to comparing findings across historical studies.

Importantly, it should be highlighted that partial ablation was used to quantify immunological changes in the post-ablative tumor microenvironment, contrasting with clinical practice where total ablation, often with additional margin, is the objective. Thus, while this study showed the ability for the immune system to clear the remaining tumor cells, that would be more representative of a worst-case focal approach whereby residual or locally infiltrative cells were inadvertently left. It is possible that, while no tumorogenic response resulted from partial ablation with either technology, surviving post-ablative cancer cells may release immunosuppressive cytokines, such as that demonstrated in (26) which may attenuate the extent of systemic immune response and benefit. This may suggest complete elimination of the tumor would further bolster overall immune response and potential abscopal effects. Future studies should determine whether complete eradication of the local tumor cell populations changes the degree and impact of immunological changes.

One aspect of this study that warrants consideration is the relatively quick (≤ 10 days) elimination of the residual primary cancer cells post-partial ablation, similar to the findings in (9, 25). Traditional adaptive immune responses may take longer to invoke anti-tumor responses. This difference may relate to the intratumoral flow cytometry Day-4 findings of rapid infiltration of innate immune cells, such as M1 macrophages and possibly natural killer cells and concurrent reduction of cells associated with tumor immunosuppression, such as M2 macrophages and mMDSCs. Thus, while adaptive immune upregulation would be required for contralateral tumor control, the innate immunity and elimination of immunosuppressive mechanisms at the ablated site may have contributed to the local tumor response here. However, caution is warranted to avoid direct extrapolation of this possibility in other tumor models, where infiltrative cancer cells beyond visible or radiologically evident tumor boundaries requires adequate surgical or ablation margins for reliable local control rates.

Another consideration for the study design is the use of contralateral inoculations four days post-ablation, rather than growing both tumors simultaneously. This approach was used due to the rapid tumor growth rate of the murine tumor model (tumor doubling time ~ 1 week), which is more aggressive than clinical tumors, making simultaneous inoculations a less translatable depiction of immune influence and abscopal response to clinical scenarios. The rapid growth rate results in a condition where several days of additional growth may make the difference between regression v. runaway growth. The inoculation of contralateral tumors four days post-ablation thus permits time for the initiation of immune activation while the contralateral tumor is established.

The examination of contralateral tumors provides insights into the systemic effects of ablation. Monitoring of contralateral tumors revealed that, although statistical significance was not achieved between the aPEF and IRE groups, mice in the aPEF group exhibited substantially smaller contralateral tumors compared to those in the IRE group at the same timepoint. This reduction in tumor size may have contributed to the prolonged median survival observed in the aPEF group by delaying the overall metastatic burden. This further implies that while primary tumor control is efficient, further optimization or combinatorial strategies such as incoroporation with adjuvant therapies, particularly immunotherapies, might be necessary to capitalize on immunostimulation to evoke meaningful systemic disease management.

Regarding the cytokines results, it is important to recognize the significant complex interrelationships between these signaling molecules, and that no specific conclusions may be drawn directly from specific changes, but rather that trends and patterns are important. aPEF was associated with higher levels of chemokines CXCL1, CXCL5, and CCL3 at 4 days post-ablation, both of which are associated with the recruitment of T cells, monocytes, and dendritic cells. IRE had lower levels of CXCL1, which may be associated with a possible suppression of the Th1 immune response, as it is a mediator of both Th1 cell activation and recruitment. These differences may partially describe the differences in intratumoral immune cell infiltration. Notably, IL-2 and IL-12—both elevated in the IRE group—are key cytokines that promote adaptive immune activation, and should be considered separately from regulatory cytokines such as IL-10 and IL-13. This highlights that IRE may induce a mixed cytokine profile with both immune-stimulatory and immunoregulatory components.

The flow cytometry data showed markedly differentiated immune responses to the Aliya-style aPEF relative to IRE. Intratumoral innate immune cell infiltration was greater for aPEF than Sham or IRE at four days post-ablation, comprising significant increases in CD3+ T-cells, B-cells, and M1 (immunostimulatory) Macrophages, as well as general trends of more natural killer and dendritic cells. It also showed reductions in immunosuppressive populations including M2 (tumor-associated macrophages, TAMs) Macrophages and mMDSCs for the aPEF group. In addition, aPEF induced pronounced systemic increases in T-cell populations and subpopulations, as well as B-cell populations, at 14 days post-ablation (**Figure 9** **).** These data indicate that the inherent, distinct differences between these technologies may cause very differentiated immune responses not only in the immediate post-ablation environment, but also lead to superior systemic adaptive immunity stimulation, highlighting the differences between these technologies. These differences in subpopulation distributions found in **Figures 3**, **4** are reflective of trends observed absolute cell counts (**Supplementary Figures 3**, **4**). Future studies with more mice should be used to determine whether the increased adaptive immune response produces better contralateral tumor control or reduces metastatic spread with metastatic tumor cell lines, such as the study performed in (25), where the primary tumor was surgically resected several days following ablation to prevent early euthanasia due to primary tumor growth. Further, while this study incorporated phenotypic profiling of immunocytes with flow cytometry, functional assessment was not performed. Assessing aspects such as activation, degranulation, and clonal expansion, exhaustion, and antigen specificity would provide valuable additional functional insight and warrants future evaluation for validating anti-tumor immunity.

A key limitation to the study reported here is the trial group sizes. This is particularly true for matching ablation size, where outcomes may be particularly sensitive to volume of ablation due to the remaining residual tumor and the amount of immune stimulation that is invoked via larger net ablation volumes. Future studies with larger cohorts would validate these findings and provide more statistically robust conclusions. Another limitation of this study is the use of the two- and three-day post-ablation timepoints to measure ablation dimensions. Preliminary work performed *(data not shown)* determined that these were suitable timepoints to capture the ablation volume from relevant cell death mechanisms prior to meaningful decreases from ablation resolution.

Another key limitation is the potential for bias in this study and ease to replicate the findings. To best control bias between technologies, the Data Integrity section details the efforts made to blind the investigators to the group for each mouse and sample. It should be noted that the objective of this study was to evaluate the immune response to Aliya-based aPEF ablation relative to typical IRE ablation, and while the specific waveform for the Aliya technology has not been disclosed, it is a commercially available system and only offers a single set of energy conditions, with replicable approaches to titrate ablation size appropriately.

This study highlights the emerging evidence that distinctions between ablation technologies can have important downstream implications on patient outcomes. Other papers have described how not all ablation is equivalent, particularly contrasting thermal modalities with non-thermal methods such as histotripsy, nsPEFs, Aliya-style advanced PEF (aPEF), and IRE, where the non-thermal methods present distinct differences in safety profile and immunostimulatory effects. Here, the effects of two distinct ablation technologies that do not rely on temperature changes are compared, showing that these technologies are different. Future investigations may evaluate the mechanistic underpinnings for these differences, particularly in the evoked cell death mechanisms and tissue structural effects, to determine the responsibility for each in causing the distinct safety and immunostimulatory properties demonstrated here.

The results presented here underscore the complex interactions between tumor ablation and immune response (**Figure 8**). Both aPEF and IRE offer valuable modalities for tumor management, with distinct advantages and limitations. Understanding these nuances is essential for integrating these technologies into comprehensive cancer intervention protocols that are both effective and minimally invasive. Since aPEF has been shown to activate the immune system by affecting both innate and adaptive immune cells, there is a strong rationale to combine aPEF with immunotherapy—a synergy previously demonstrated in earlier publications (9, 25), but was not evaluated here. Future studies may explore methods to further enhance their efficacy, particularly through combinations with immunotherapies, to fully exploit their potential in oncological applications.

## 5 Conclusion

This study examined the immune response and tumor control benefits invoked from size-matched partial ablation of two different technologies, IRE and aPEF, that have both been shown previously to induce beneficial immunostimulatory responses to their ablation. The results demonstrate a clear delineation in downstream immunostimulatory response to the aPEF ablation relative to IRE, including differentiated cytokine profiles and increased immune cell infiltration in the tumor microenvironment in the short-term, followed by increased adaptive circulating immune cell populations for aPEF in the long-term. These changes were associated with improved local and distant tumor control and mouse survival. This study demonstrates that aPEF appears to invoke a more favorable anti-tumor immune response than IRE.

## Data Availability

The original contributions presented in the study are included in the article/**Supplementary Material**. Further inquiries can be directed to the corresponding author.
