# Supplementary material for: Evaluating the immune response in a murine cancer model between irreversible electroporation and an advanced biphasic pulsed electric field technology
Source: Front Oncol. 2025 Jun 24;15:1592610. doi: 10.3389/fonc.2025.1592610 (PMC12235187; doi:10.3389/fonc.2025.1592610)
Supplement: Supplementary file 1 [file DataSheet1.docx]

# **Supplementary Data**

# **Supplementary Figures**

### **Supplementary Figure1*.***


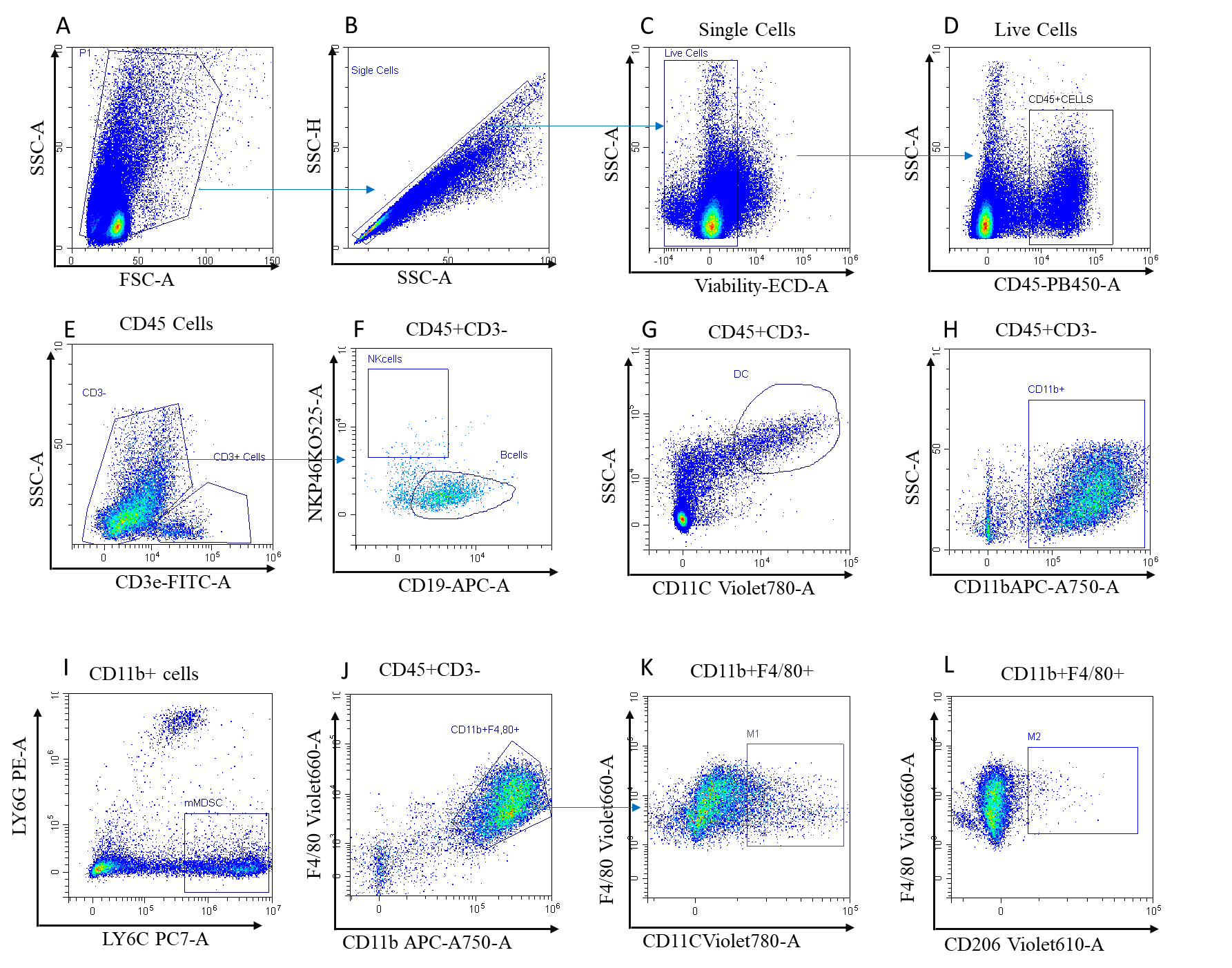


### **Supplementary Figure1*.* Gating Strategy for Immune Cell Subset Identification in tumor cells 4 days post-ablation.** Panels (A–L) illustrate the sequential gating strategy used to identify and characterize immune cell subsets from a mixed population using flow cytometry. (A) Forward scatter area (FSC-A) vs. side scatter area (SSC-A) plot to identify the overall cell population. (B) FSC-H vs. FSC-A plot to gate on singlets and exclude doublets. (C) SSC-A vs. viability dye (Viability-ECD-A) to isolate live cells. (D) CD45 expression to select leukocytes (CD45+). (E) CD3 expression to distinguish T cells (CD3+) from other immune cells (CD3−).(F) CD45+CD3− cells analyzed for NK cells (NKp46+) and B cells (CD19+). (G) CD45+CD3− cells further gated on CD11c expression to identify dendritic cells (DC). (H) CD45+CD3− cells gated on CD11b expression to identify additional myeloid populations.Panels (I–L): Macrophage and Myeloid Subset Gating Strategy. (I) CD11b+ cells gated for Ly6G vs. Ly6C to identify monocytic myeloid-derived suppressor cells (mMDSCs; Ly6C+Ly6G−). (J) CD45+CD3−CD11b+F4/80+ cells selected as macrophages. (K) Macrophages (CD11b+F4/80+) gated on CD11c expression to identify M1 macrophages (pro-inflammatory). (L) Macrophages (CD11b+F4/80+) gated on CD206 expression to identify M2 macrophages (anti-inflammatory).

**Supplementary Figure 2.**
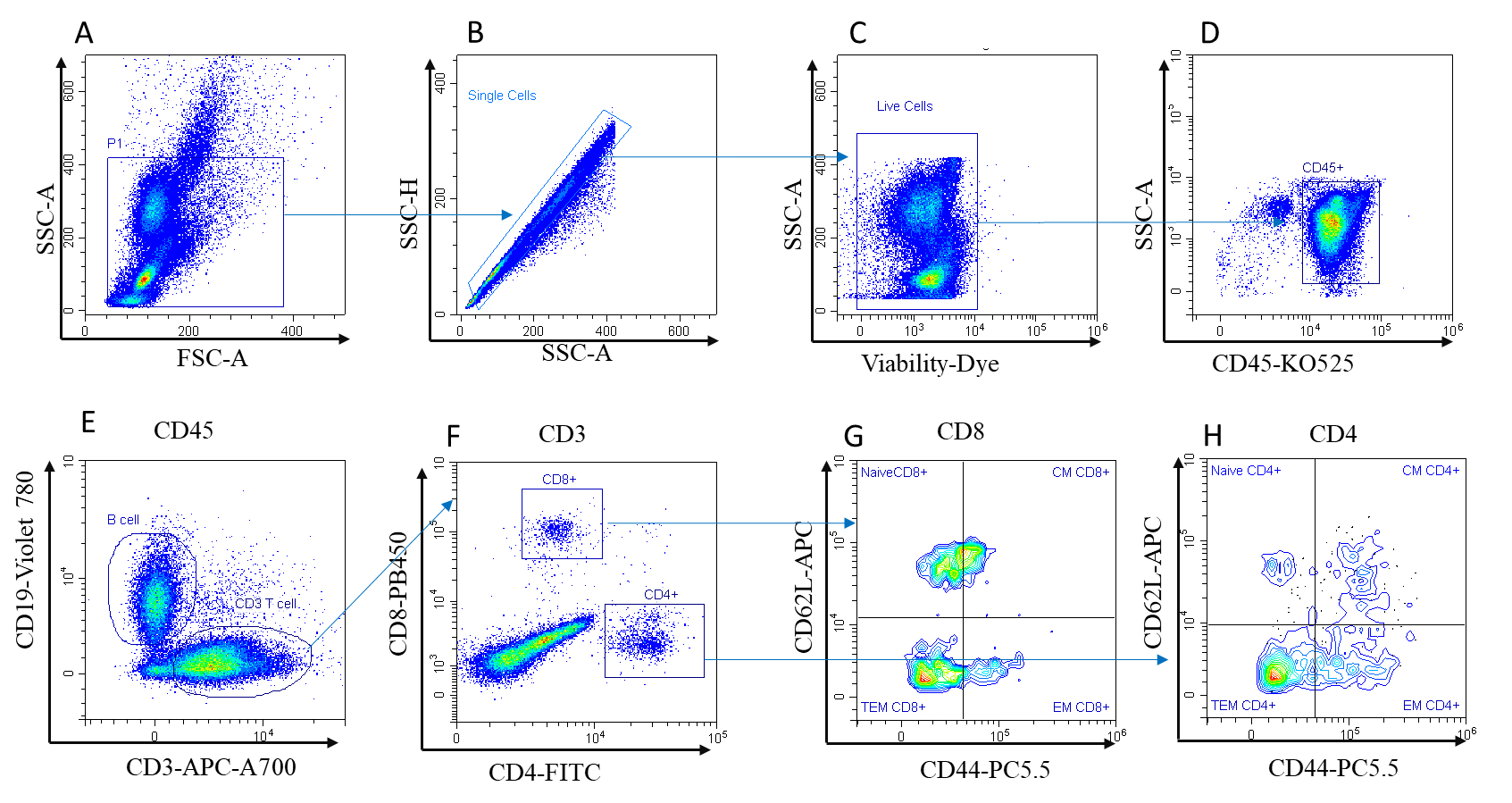


**Supplementary Figure 2. Gating Strategy for Identifying Immune Cell Subsets in Blood 14 days post-ablation**. Panels (A–H) illustrate the sequential gating strategy used to identify and characterize immune cell subsets from a mixed population using flow cytometry. (A) Forward scatter area (FSC-A) vs. side scatter area (SSC-A) plot to identify the overall cell population. (B) FSC-H vs. FSC-A plot to gate on singlets and exclude doublets. (C) SSC-A vs. viability dye to exclude dead cells and select live cells. (D) CD45 expression to identify leukocytes. (E) CD45 vs. CD3 plot to distinguish B cells (CD19+) and T cells (CD3+). (F) CD3 vs. CD4/CD8 to classify T cell subsets into CD4+ and CD8+ populations. (G) CD44 vs. CD62L expression on CD8+ T cells to identify their activation states: naive (CD62L+CD44−), central memory (CM, CD62L+CD44+), effector memory (EM, CD62L−CD44+), and terminal effector (TEM, CD62L−CD44−).(H) CD44 vs. CD62L expression on CD4+ T cells to identify their activation states as naive, CM, EM, or TEM analogously to CD8+ T cells.

**Supplementary Figure 3.**


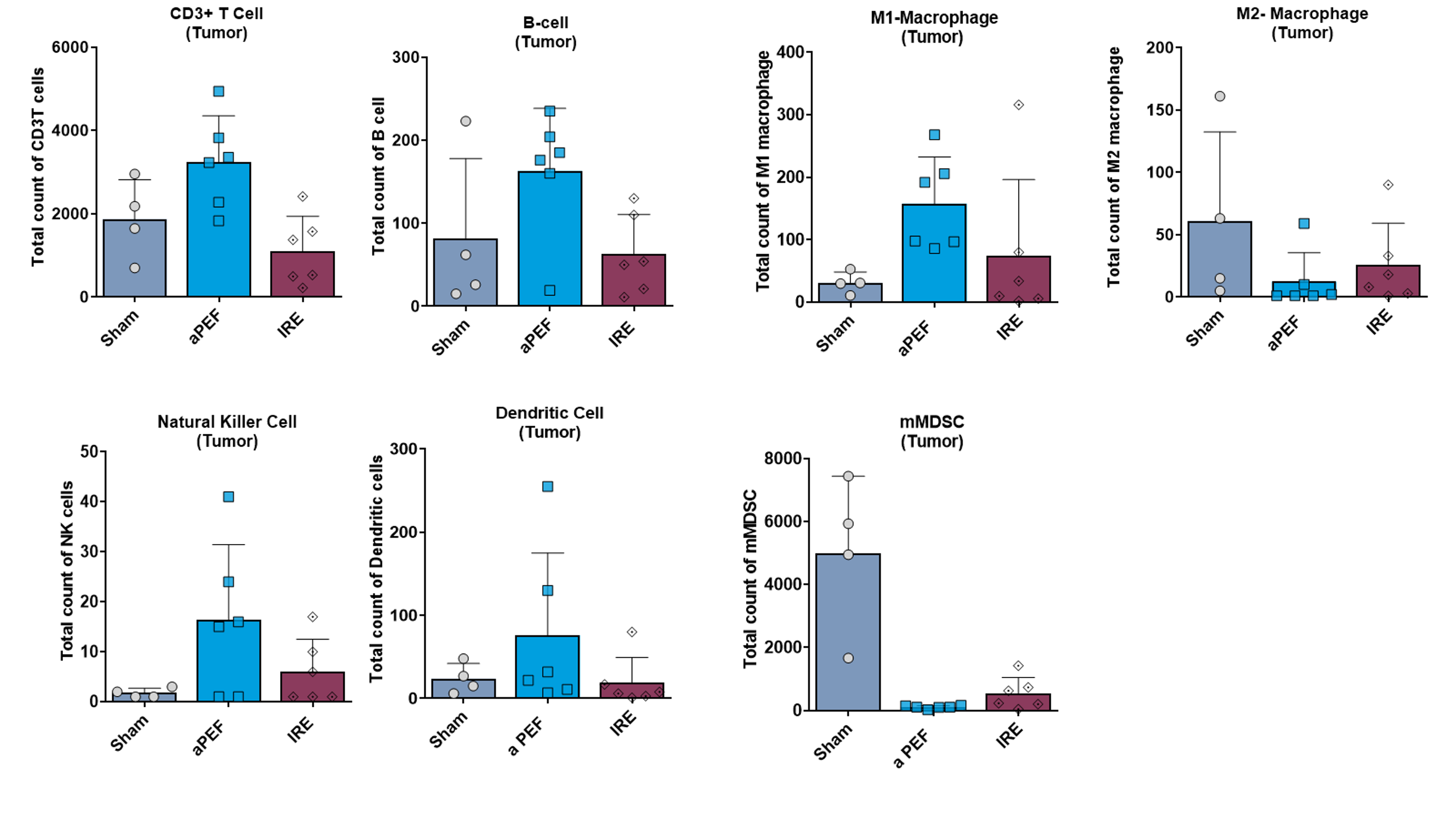


**Supplementary Figure 3. Intratumoral Immune Cell Counts Following Aliya PEF and IRE Treatments.** Graphs depict the total counts of CD3+ T cells, B cells, M1-macrophage, M2 macrophage, Natural Killer cells, Dendritic cells and mMDSC cells, enumerated intratumorally from mice subjected to Sham, aPEF, or IRE treatments.

**Supplementary Figure 4.**


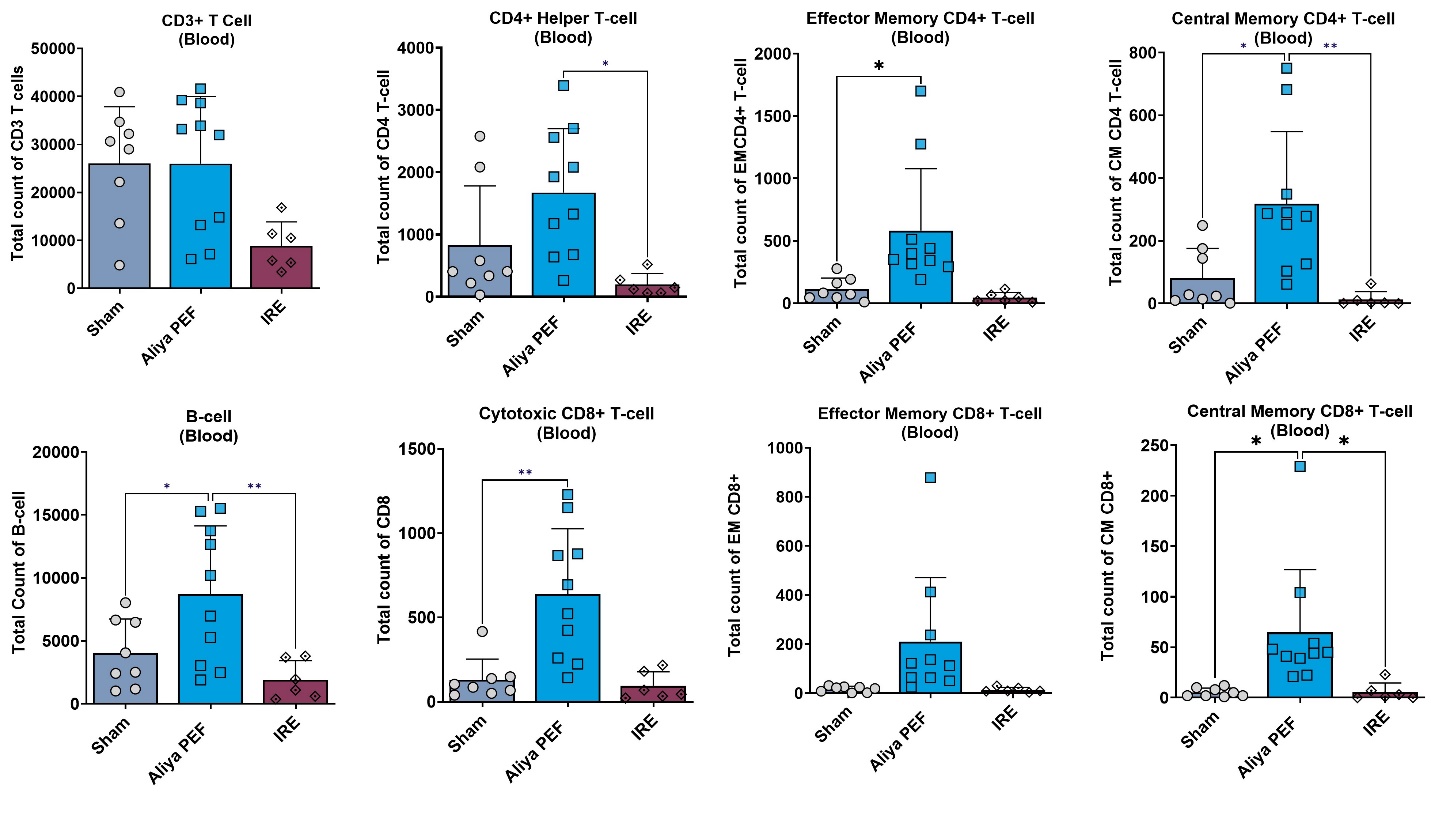


**Supplementary Figure 4. Systemic Immune Cell Counts Following Aliya PEF and IRE Treatments.** Graphs depict the total counts of CD3+ T cells, CD4+ helper T cells, effector memory CD4+ T cells, central memory CD4+ T cells, B cells, cytotoxic CD8+ T cells, effector memory CD8+ T cells, and central memory CD8+ T cells in peripheral blood samples from mice subjected to Sham, aPEF, or IRE treatments

# **Supplementary Tables**

**Supplementary Table 1.** Cell surface markers used for characterization of immune cell types in flow cytometric analysis.

| **Antibody** | **Vendor and Catalog number** |
| --- | --- |
| Anti-mouse CD45 | Biolegend, 103126 |
| Anti-Mouse CD3ε | Biolegend, 100216 |
| Anti-Mouse CD8a | Biolegend, 100753 |
| Anti-Mouse CD4 | BD Biosciences, 130308 |
| Anti-mouse CD44 | Biolegend, 103032 |
| Anti-Mouse CD62L | BD Biosciences, 553152 |
| Anti-Mouse NKp46 | Biolegend,137604 |
| Anti-Mouse CD19 | Biolegend,115512 |
| Anti-mouse/human CD11b | Biolegend, 101226 |
| Anti-mouse F4/80 | Biolegend, 123149 |
| Anti-mouse CD11c | Biolegend, 117339 |
| Anti-mouse CD206 | Biolegend, C068C2 |
| Anti-mouse Ly-6C | Biolegend, 128017 |
| Anti-mouse Ly-6G | Biolegend, 164503 |
| Anti-mouse MHC Class II | Biolegend, 116424 |
| Propidium iodide | Biolegend, 421301 |

**Supplementary Table 2.** Marker Profiles for Identifying Immune Cell Populations in tumor

| **Population Name** | **Markers** |
| --- | --- |
| **Leukocytes** | CD45+ |
| **T Cells** | CD45+, CD3+ |
| **Non-T Cells** | CD45+, CD3− |
| **B Cells** | CD45+, CD3−, CD19+ |
| **NK Cells** | CD45+, CD3−, NKp46+ |
| **Dendritic Cells (DC)** | CD45+, CD3−, CD11c+ |
| **Myeloid Cells** | CD45+, CD3−, CD11b+ |
| **Monocytic MDSCs (mMDSCs)** | CD45+, CD3−, CD11b+, Ly6C+, Ly6G− |
| **Macrophages** | CD45+, CD3−, CD11b+, F4/80+ |
| **M1 Macrophages** | CD45+, CD3−, CD11b+, F4/80+, CD11c+ |
| **M2 Macrophages** | CD45+, CD3−, CD11b+, F4/80+, CD206+ |

**Supplementary Table 3.** Marker Profiles for Identifying Immune Cell Populations in blood

| **Population Name** | **Markers** |
| --- | --- |
| **Leukocytes** | CD45+ |
| **B-cells** | CD45+, CD3-, CD19+ |
| **T-cells** | CD45+, CD3+ |
| **CD4+ T-cells** | CD45+, CD3+, CD4+ |
| **CD8+ T-cells** | CD45+, CD3+, CD8+ |
| **Central Memory CD8+** | CD45+, CD3+, CD8+, CD62L-, CD44+ |
| **Effector Memory CD8+** | CD45+, CD3+, CD8+, CD62L-, CD44+ |
| **Central Memory CD4+** | CD45+, CD3+, CD4+, CD62L+, CD44+ |
| **Effector Memory CD4+** | CD45+, CD3+, CD4+, CD62L-, CD44+ |

**Supplementary Table 4.** Tumor Cytokine Levels (Fluorescence intensity) and Multiple t-test Analysis 4 Days Post-Ablation

| **Cytokines** | **Mean of Sham (n=4)** | **SD of Sham** | **Mean of aPEF (n=8)** | **SD of aPEF** | **p-value Sham v. aPEF** | | **Mean of IRE (n=8)** | **SD of IRE** | **p-value Sham v. IRE** | **p-value aPEF v. IRE** | **Significance between aPEF v IRE** |
| --- | --- | --- | --- | --- | --- | --- | --- | --- | --- | --- | --- |
| **Eotaxin** | 6168 | 2908 | 1714 | 1773 | 0.016 | 6420 | | 2500 | 0.878 | 0.002 | YES |
| **G-CSF** | 333.6 | 120 | 285.3 | 180 | 0.653 | 422 | | 366 | 0.655 | 0.42 | - |
| **GM-CSF** | 267.2 | 106 | 80.22 | 49 | 0.005 | 135.1 | | 62 | 0.02 | 0.101 | - |
| **IFNγ** | 87.28 | 10 | 49.88 | 14 | 0.002 | 83.63 | | 53 | 0.896 | 0.156 | - |
| **IL-1α** | 101 | 33 | 103.3 | 48 | 0.935 | 336.5 | | 420 | 0.3 | 0.205 | - |
| **IL-1β** | 61.03 | 23 | 30.3 | 11 | 0.02 | 52.33 | | 23 | 0.546 | 0.051 | YES |
| **IL-2** | 83.78 | 13 | 19.58 | 2 | 0 | 62.3 | | 32 | 0.229 | 0.007 | YES |
| **IL-3** | 77.08 | 30 | 45.48 | 25 | 0.108 | 56.51 | | 29 | 0.274 | 0.471 | - |
| **IL-4** | 767.9 | 965 | 153 | 252 | 0.165 | 363.1 | | 332 | 0.294 | 0.221 | - |
| **IL-5** | 14.08 | 1 | 11.42 | 2 | 0.021 | 11.18 | | 2 | 0.007 | 0.79 | - |
| **IL-6** | 255.1 | 73 | 209 | 113 | 0.494 | 209.4 | | 62 | 0.282 | 0.994 | - |
| **IL-7** | 44.78 | 11 | 24.6 | 2 | 0.002 | 33.3 | | 8 | 0.063 | 0.022 | YES |
| **IL-9** | 44.35 | 12 | 32.47 | 2 | 0.035 | 41.61 | | 14 | 0.744 | 0.14 | - |
| **IL-10** | 48.7 | 13 | 25.93 | 3 | 0.002 | 48.6 | | 24 | 0.994 | 0.039 | YES |
| **IL-12p40** | 121.9 | 22 | 64.45 | 10 | 0 | 85.93 | | 22 | 0.023 | 0.046 | YES |
| **IL-12p70** | 15.95 | 1 | 14.82 | 2 | 0.406 | 14.96 | | 2 | 0.313 | 0.896 | - |
| **IL-13** | 73.68 | 24 | 36.87 | 9 | 0.008 | 57.76 | | 21 | 0.265 | 0.045 | YES |
| **IL-15** | 81.9 | 19 | 77.55 | 46 | 0.865 | 69.3 | | 18 | 0.281 | 0.648 | - |
| **IL-17** | 25.15 | 4 | 85.82 | 49 | 0.042 | 55.68 | | 38 | 0.147 | 0.217 | - |
| **CXCL10** | 3919 | 1480 | 2496 | 864 | 0.088 | 3003 | | 1096 | 0.249 | 0.369 | - |
| **CXCL1** | 656.6 | 171 | 1020 | 517 | 0.218 | 480.9 | | 271 | 0.269 | 0.026 | YES |
| **LIF** | 553.7 | 260 | 275.6 | 87 | 0.038 | 356.4 | | 201 | 0.175 | 0.379 | - |
| **CXCL5** | 108.7 | 11 | 299.3 | 186 | 0.079 | 109.4 | | 30 | 0.967 | 0.014 | YES |
| **M-CSF** | 105.5 | 19 | 74.02 | 21 | 0.044 | 98.2 | | 41 | 0.748 | 0.214 | - |
| **CCL2** | 654.2 | 87 | 462 | 217 | 0.136 | 425.5 | | 206 | 0.063 | 0.753 | - |
| **CXCL9** | 8306 | 4739 | 4199 | 2811 | 0.12 | 7782 | | 2118 | 0.791 | 0.018 | YES |
| **CCL3** | 67.4 | 17 | 727.4 | 604 | 0.065 | 235 | | 243 | 0.208 | 0.056 | YES |
| **CCL4** | 117.4 | 26 | 486.8 | 344 | 0.069 | 246.5 | | 195 | 0.227 | 0.122 | - |
| **CXCL2** | 505.8 | 225 | 5670 | 3721 | 0.026 | 2910 | | 3771 | 0.242 | 0.198 | - |
| **RANTES** | 126.4 | 30 | 58.07 | 17 | 0.002 | 94.26 | | 47 | 0.246 | 0.099 | - |
| **TNFα** | 115.4 | 34 | 97.6 | 56 | 0.587 | 73.58 | | 27 | 0.044 | 0.305 | - |
| **VEGF** | 5988 | 1729 | 3974 | 1847 | 0.122 | 3578 | | 1369 | 0.024 | 0.653 | - |

**Supplementary Table 5.** Serum Cytokine Levels (fluorescence intensity) and Statistical Analysis 4 Days Post-Ablation

| **Cytokines** | **Mean of Sham (n=4)** | **SD of Sham** | **Mean of aPEF (n=6)** | **SD of aPEF** | **p-value Sham V aPEF** | **Mean of IRE (n=8)** | **SD of IRE** | **p-value Sham v. IRE** | **p-value aPEF v. IRE** | **Significance between aPEF & IRE** |
| --- | --- | --- | --- | --- | --- | --- | --- | --- | --- | --- |
| **Eotaxin** | 4829 | 1555 | 6315 | 1809 | 0.217 | 6663 | 840 | 0.022 | 0.637 | - |
| **G-CSF** | 441.4 | 203 | 135.6 | 59 | 0.007 | 187.4 | 140 | 0.028 | 0.416 | - |
| **GM-CSF** | 9.65 | 3 | 9.383 | 2 | 0.868 | 9.038 | 1 | 0.6 | 0.715 | - |
| **IFNγ** | 18.28 | 3 | 17.53 | 3 | 0.725 | 15.43 | 3 | 0.145 | 0.218 | - |
| **IL-1α** | 65 | 27 | 110.7 | 31 | 0.043 | 132.8 | 57 | 0.051 | 0.411 | - |
| **IL-1β** | 11.2 | 4 | 9.85 | 1 | 0.45 | 7.788 | 1 | 0.056 | 0.012 | YES |
| **IL-2** | 7.825 | 2 | 7.967 | 1 | 0.895 | 7.85 | 1 | 0.974 | 0.847 | - |
| **IL-3** | 13.38 | 2 | 10.72 | 2 | 0.044 | 13.58 | 7 | 0.959 | 0.369 | - |
| **IL-4** | 14.13 | 1 | 11.23 | 2 | 0.015 | 12.83 | 2 | 0.27 | 0.164 | - |
| **IL-5** | 48.95 | 18 | 73.88 | 23 | 0.105 | 54.39 | 28 | 0.738 | 0.194 | - |
| **IL-6** | 163.8 | 103 | 68.17 | 15 | 0.05 | 100.5 | 44 | 0.157 | 0.112 | - |
| **IL-7** | 187.5 | 289 | 17.88 | 6 | 0.176 | 40.54 | 71 | 0.186 | 0.457 | - |
| **IL-9** | 38.7 | 41 | 15.08 | 8 | 0.192 | 27.54 | 26 | 0.57 | 0.28 | - |
| **IL-10** | 16.2 | 2 | 14.25 | 1 | 0.131 | 14.7 | 2 | 0.26 | 0.624 | - |
| **IL-12p40** | 24.28 | 4 | 17.63 | 5 | 0.058 | 19.51 | 12 | 0.454 | 0.718 | - |
| **IL-12p70** | 10.75 | 2 | 9.45 | 1 | 0.172 | 10.29 | 2 | 0.711 | 0.37 | - |
| **IL-13** | 18.65 | 7 | 14.82 | 5 | 0.312 | 17.11 | 4 | 0.616 | 0.335 | - |
| **IL-15** | 212.5 | 215 | 35.85 | 14 | 0.072 | 108.4 | 236 | 0.477 | 0.471 | - |
| **IL-17** | 17.13 | 3 | 13.92 | 3 | 0.124 | 16.16 | 7 | 0.8 | 0.471 | - |
| **CXCL10** | 1406 | 65 | 1144 | 216 | 0.049 | 1364 | 188 | 0.679 | 0.065 | - |
| **CXCL1** | 42.5 | 16 | 55.73 | 6 | 0.106 | 69.55 | 26 | 0.091 | 0.233 | - |
| **LIF** | 313.1 | 445 | 30.88 | 10 | 0.147 | 53.14 | 85 | 0.125 | 0.54 | - |
| **CXCL5** | 3680 | 1501 | 3621 | 1909 | 0.96 | 3506 | 1577 | 0.858 | 0.904 | - |
| **M-CSF** | 13.03 | 2 | 11.05 | 1 | 0.06 | 12.75 | 5 | 0.923 | 0.457 | - |
| **CCL2** | 11.45 | 3 | 11.97 | 3 | 0.792 | 10.76 | 2 | 0.674 | 0.358 | - |
| **CXCL9** | 2278 | 709 | 1656 | 416 | 0.115 | 2118 | 875 | 0.759 | 0.258 | - |
| **CCL3** | 10.75 | 0 | 10.43 | 1 | 0.513 | 9.638 | 2 | 0.195 | 0.284 | - |
| **CCL4** | 34.33 | 8 | 30.18 | 5 | 0.329 | 33.64 | 8 | 0.891 | 0.358 | - |
| **CXCL2** | 17.65 | 2 | 17.57 | 2 | 0.953 | 16.95 | 4 | 0.746 | 0.732 | - |
| **RANTES** | 40.45 | 12 | 25.68 | 7 | 0.04 | 27.54 | 15 | 0.176 | 0.792 | - |
| **TNFα** | 20 | 6 | 16.85 | 2 | 0.271 | 13.55 | 3 | 0.028 | 0.015 | YES |
| **VEGF** | 23.63 | 2 | 27.77 | 7 | 0.303 | 28.35 | 7 | 0.248 | 0.884 | - |
